# Cerebellar Perfusion Changes After Repetitive Deep Transcranial Magnetic Stimulation in Parkinson’s Disease: A Five-Patient Case Series and Literature Review

**DOI:** 10.3390/diagnostics16162619

**Published:** 2026-08-18

**Authors:** In-Uk Song, Yong An Chung, Byung Seok Kim, Seunghee Na, Sonya Young Joo Park

**Affiliations:** 1 Department of Neurology, Incheon St. Mary’s Hospital, College of Medicine, The Catholic University of Korea, Seoul 06591, Republic of Korea; siuy70@gmail.com (I.-U.S.); bskim41979@gmail.com (B.S.K.); seunghee.na@gmail.com (S.N.); 2 Department of Nuclear Medicine, Incheon St. Mary’s Hospital, College of Medicine, The Catholic University of Korea, Seoul 06591, Republic of Korea; 3 Department of Nuclear Medicine, Yeouido St. Mary’s Hospital, College of Medicine, The Catholic University of Korea, Seoul 06591, Republic of Korea; park@catholic.ac.kr

**Keywords:** Parkinson’s disease, deep transcranial magnetic stimulation, regional cerebral blood flow, supplementary motor area

## Abstract

**Background and Clinical Significance:** Parkinson’s disease (PD) is a progressive neurodegenerative disorder for which nonpharmacological neuromodulatory approaches are being explored as adjunctive strategies. Deep transcranial magnetic stimulation (dTMS) using an H-coil can engage broader and deeper cortical networks than conventional focal coils, although anatomically distant structures are expected to be influenced through network modulation rather than direct electromagnetic stimulation. **Case Presentation:** We describe a five-patient exploratory case series evaluating clinical measures and cerebral perfusion before and three months after high-frequency dTMS targeting the supplementary motor area (SMA). Clinical outcomes included the Unified Parkinson’s Disease Rating Scale (UPDRS) Parts I-IV, Non-Motor Symptoms Scale (NMSS), Hoehn–Yahr stage, and Timed Up and Go test. Antiparkinsonian medication regimens and doses remained unchanged during the three-month follow-up. Cerebral perfusion was assessed using single-photon emission computed tomography (SPECT) and statistical parametric mapping. No clinical outcome reached nominal statistical significance at follow-up; given the sample size, this should be interpreted as limited statistical power rather than evidence of no effect. At an exploratory voxel-wise threshold of *p* < 0.001, uncorrected, SPECT identified a 28-voxel cluster of increased regional cerebral blood flow in the right cerebellar cortex (peak MNI coordinates: 22, −38, −44; t = 5.96; z = 3.11). **Conclusions:** These observations are hypothesis-generating and may reflect network-level modulation of SMA–cerebellar circuitry. Because only five patients were included, no sham-controlled arm was available, and the imaging analysis used an uncorrected exploratory threshold, causal or efficacy claims cannot be made. Larger sham-controlled studies with standardized dose-to-imaging timing, formal neuropsychological assessment, individualized targeting, and prespecified corrected imaging analyses are warranted.

## 1. Introduction

Parkinson’s disease (PD) is a widespread neurodegenerative disorder characterized by bradykinesia, rigidity, resting tremor, and postural instability [1]. Levodopa provides effective symptomatic relief, but motor fluctuations and treatment-refractory symptoms commonly emerge as the disease progresses [2]. Repetitive transcranial magnetic stimulation (rTMS) is a noninvasive neuromodulation technique that can alter cortical excitability and connected motor networks through electromagnetic induction [3,4,5,6]. Clinical studies in PD have reported heterogeneous motor effects, reflecting differences in stimulation frequency, target, coil geometry, treatment duration, and sample characteristics [4,7,8]. The SMA is of particular interest because it participates in internally generated movement and gait control, and randomized studies have shown that SMA-directed rTMS can modify motor outcomes in PD [9,10,11].

Functional neuroimaging such as positron emission tomography (PET) and single-photon emission computed tomography (SPECT) can provide objective information about treatment-associated changes in cerebral physiology [12,13,14]. Brain perfusion SPECT measures regional cerebral blood flow (rCBF), which is coupled to regional neuronal activity and can therefore complement clinical outcome measures. Nevertheless, imaging studies specifically examining perfusion changes after SMA-targeted deep TMS in PD remain scarce, leaving an important mechanistic gap between clinical neuromodulation studies and network-level physiological effects.

Most conventional rTMS studies use circular or figure-of-eight coils that produce relatively focal electric fields concentrated in the superficial cortex. Modeling and phantom measurements have shown that the figure-of-eight coil produces suprathreshold fields focally at relatively shallow depths, whereas H-coils trade some focality for broader and deeper field penetration [15,16,17,18]. This characteristic makes the H-coil potentially useful when the therapeutic hypothesis involves distributed medial frontal and motor networks rather than a single superficial cortical point. Importantly, however, the H-coil should not be considered capable of directly stimulating anatomically distant structures such as the cerebellum from an SMA scalp position; any cerebellar change is more plausibly interpreted as downstream modulation through connected cortico-ponto-cerebellar and cerebello-thalamo-cortical circuits. H-coil studies in PD have provided preliminary and sham-controlled evidence supporting the feasibility of this approach [4,19,20]. We therefore used an H1-coil to explore whether high-frequency dTMS targeting the SMA is associated with measurable changes in cerebral perfusion in PD.

## 2. Case Presentation and Clinical Assessment

Five patients with PD were included in this exploratory case series. PD was clinically diagnosed based on the presence of at least two of the three cardinal motor features: resting tremor, rigidity, and bradykinesia. All patients also showed reduced dopamine transporter uptake in the posterior putamen on I-123 N-fluoropropyl-2β-carbomethoxy-3β-(4-iodophenyl)nortropane (FP-CIT) PET. Exclusion criteria included significant medical or psychiatric illness, a history of seizure, abnormal electroencephalography findings, and intracranial metal objects or stimulators that could pose a safety risk during dTMS. Patients with clinical features suggestive of atypical parkinsonism, delirium, amnestic disorders, or cerebrovascular lesions on magnetic resonance imaging were excluded, as were patients taking psychotropic medications. Marked cognitive dysfunction was excluded on the basis of clinical history, neurologic interview, ability to understand study procedures, and ability to complete the assessment protocol. A uniform standardized cognitive screening instrument (e.g., MMSE or MoCA) with a prespecified cutoff was not prospectively administered to all five participants; this is explicitly acknowledged as a methodological limitation rather than implying that a standardized neuropsychological battery was used.

A comprehensive clinical assessment, including medical history and neurological examination, was performed by board-certified neurologists. Disease severity, motor function, activities of daily living, motor complications, and non-motor symptoms were assessed using the Hoehn–Yahr (HY) stage, UPDRS Parts I-IV, the Non-Motor Symptoms Scale (NMSS), and the Timed Up and Go (TUG) test [21,22]. For the TUG, the participant was timed while rising from a standard chair, walking 3 m at a comfortable and safe pace, turning, walking back to the chair, and sitting down; the total time was recorded in seconds [22]. Clinical assessments and SPECT imaging were performed at baseline and three months after dTMS. Dedicated depression, anxiety, and apathy instruments and a formal neuropsychological battery were not prospectively administered to all participants. Antiparkinsonian medication regimens and doses remained unchanged from baseline through the three-month follow-up; consequently, each patient’s three-month levodopa equivalent daily dose (LEDD) was identical to the baseline value reported in Table 1. The exact drug-by-drug regimen was not retained in the analytic dataset, and the interval between the most recent dopaminergic dose and each SPECT acquisition was not prospectively standardized. Because acute levodopa can alter regional cerebral blood flow [23], dose-to-imaging timing remains a potential imaging confounder despite the stable medication regimen.

Changes in HY stage, UPDRS Parts I–IV, NMSS, and TUG from baseline to the three-month follow-up were evaluated using two-sided paired Wilcoxon signed-rank tests. During revision, the original numerical *p*-values were rechecked against the individual paired data and recalculated consistently using the paired analysis. Because this was an exploratory five-patient series, no formal correction for the seven clinical comparisons was applied, and *p*-values are interpreted descriptively. To complement *p*-values, paired-sample standardized effect sizes (Cohen’s dz, calculated as the mean within-patient change divided by the standard deviation of the within-patient changes) are reported in Table 2. With *n* = 5, both hypothesis tests and effect-size estimates are imprecise; failure to reach statistical significance should not be interpreted as evidence of no biological or clinical effect. No correlations between clinical score changes and the SPECT cluster were performed because patient-level regional perfusion values from the identified cluster were not prespecified for extraction and such correlations would be highly unstable in a sample of five. The study was approved by the Institutional Review Board, and all participants provided written informed consent.

### 2.1. Transcranial Magnetic Stimulation

Within four weeks after baseline clinical evaluation and SPECT imaging, all participants received dTMS targeting the left SMA three times per week for two weeks (six sessions in total). Stimulation was administered using a BrainsWay H1-coil with either a Magstim Rapid2 stimulator (Magstim Company, Spring Gardens, UK) or a BrainsWay stimulator (BrainsWay, Jerusalem, Israel). The intended stimulation target was the left SMA for all participants and was not switched according to the clinically more affected side. The H1-coil produces a broad electric-field distribution, so bilateral and network-level engagement may occur even though the scalp-based intended target was left-sided. The coil is mounted in a helmet that allows cooling during stimulation and accommodates individual variability in head shape [16].

**Figure 1:**
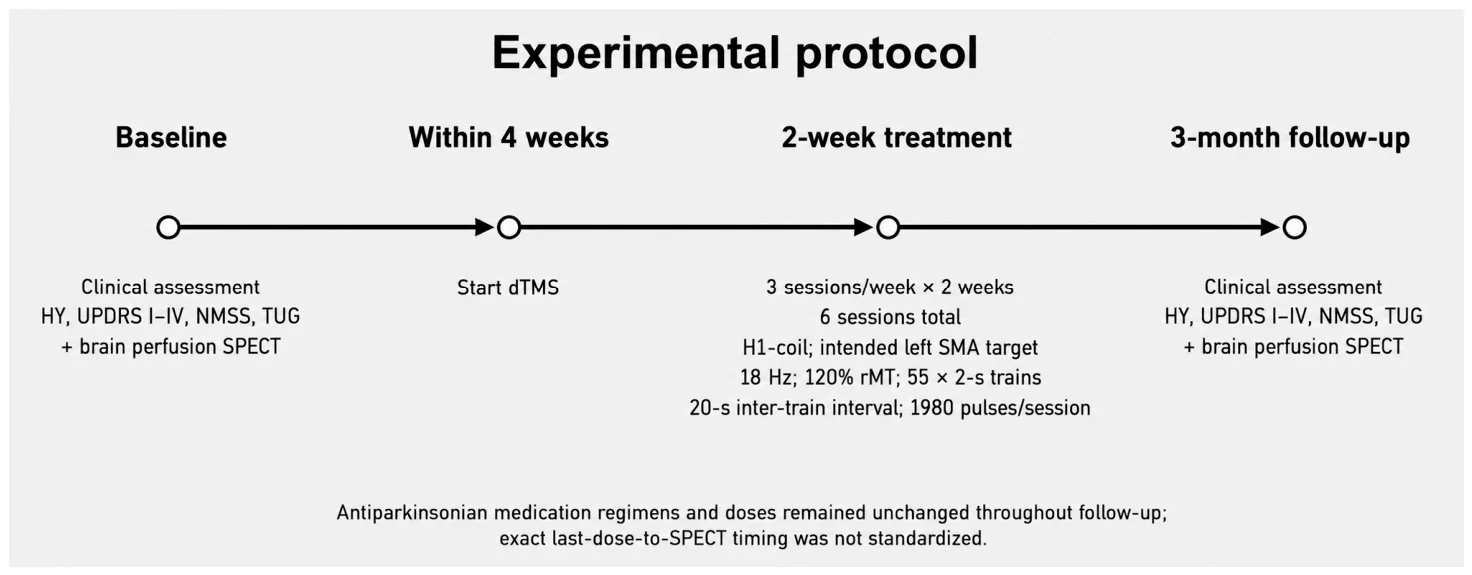
Experimental protocol. Baseline clinical assessment (HY stage, UPDRS Parts I-IV, NMSS, and TUG) and brain perfusion SPECT were followed within four weeks by six H1-coil dTMS sessions (three sessions per week for two weeks). The intended scalp-based target was the left SMA. Clinical assessment and SPECT were repeated at three months. Antiparkinsonian medication regimens and doses remained unchanged throughout follow-up, although exact last-dose-to-SPECT timing was not prospectively standardized. dTMS, deep transcranial magnetic stimulation; HY, Hoehn–Yahr; NMSS, Non-Motor Symptoms Scale; rMT, resting motor threshold; SMA, supplementary motor area; SPECT, single-photon emission computed tomography; TUG, Timed Up and Go; UPDRS, Unified Parkinson’s Disease Rating Scale.

The resting motor threshold (rMT) was determined with the H1-coil positioned over the left primary motor cortex (M1) by identifying the minimum stimulation intensity required to elicit a visible motor response in the contralateral hand muscles. After rMT determination, the coil was moved 2 cm anterior to the M1 motor hotspot to approximate the SMA. Individual MRI-guided neuronavigation was not used; therefore, the scalp-based position represented an estimated SMA target and may have differed anatomically among participants. The dTMS protocol consisted of 18 Hz stimulation at 120% of rMT, 55 trains of 2 s each, a 20 s inter-train interval, and 1980 pulses per session. Each session lasted approximately 20 min. The overall study sequence and timing of assessments are summarized in Figure 1.

### 2.2. SPECT Acquisition and Analysis

Brain SPECT was performed at baseline and at the three-month follow-up using a dual-headed gamma camera (Discovery NM630, GE Healthcare, Milwaukee, WI, USA) equipped with a low-energy fan-beam collimator. Images were obtained 40 min after intravenous injection of 555–740 MBq of technetium-99m ethyl cysteinate dimer.

Image preprocessing and voxel-wise analyses were performed using Statistical Parametric Mapping 12 (SPM12; Wellcome Trust Centre for Neuroimaging, London, UK) implemented in MATLAB R2017b (MathWorks, Natick, MA, USA). A paired *t*-test was used to assess within-subject changes in rCBF between baseline and follow-up. Given the exploratory nature and very small sample size, the voxel-wise threshold was set at *p* < 0.001, uncorrected. Cluster extent, peak t- and z-values, and Montreal Neurological Institute (MNI) coordinates were recorded descriptively from the SPM output. No family-wise error (FWE) or false-discovery rate (FDR) correction was applied; accordingly, the imaging result is interpreted as an exploratory, uncorrected finding rather than confirmatory whole-brain statistical evidence.

## 3. Results

**Figure 2:**
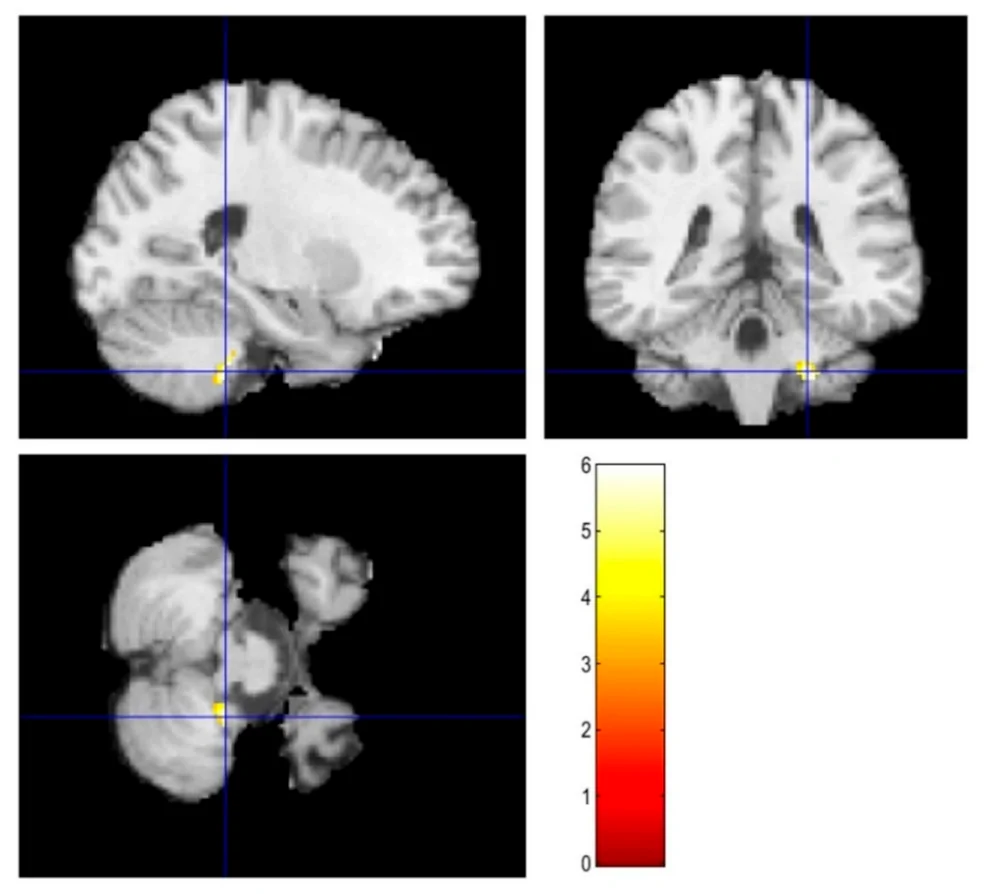
Exploratory changes in relative regional cerebral blood flow (rCBF) at the three-month follow-up compared with baseline. Increased rCBF is shown in the right cerebellar cortex at a voxel-wise threshold of *p* < 0.001, uncorrected. The cluster contained 28 voxels, with a peak at MNI coordinates (22, −38, −44) (t = 5.96; z = 3.11; *p* < 0.001). No FWE or FDR correction was applied. The color bar represents voxel-level t-values.

All five patients completed six dTMS sessions over two weeks. Demographic and individual clinical characteristics are summarized in Table 1. Antiparkinsonian medication regimens and doses were unchanged during the three-month follow-up; therefore, follow-up LEDD values were identical to baseline for all five patients. None of the seven clinical outcomes reached nominal statistical significance at follow-up (HY, *p* = 1.000; UPDRS Part I, *p* = 0.750; UPDRS Part II, *p* = 1.000; UPDRS Part III, *p* = 0.125; UPDRS Part IV, *p* = 1.000; NMSS, *p* = 0.750; TUG, *p* = 0.125). Because the study included only five patients, these nonsignificant *p*-values should be interpreted as inconclusive rather than as evidence that dTMS had no clinical effect. Descriptive paired effect sizes are shown in Table 2. Mean NMSS decreased from 15.20 ± 13.72 to 10.50 ± 8.38, whereas mean UPDRS Part I changed from 0.20 ± 0.45 to 0.80 ± 1.30; neither change was statistically significant. Individual motor responses were heterogeneous: UPDRS Part III decreased in Cases 1–4 but was unchanged in Case 5, whereas UPDRS Part IV improved in Cases 1 and 2, was unchanged in Cases 3 and 4, and worsened in Case 5. In the SPECT analysis, the exploratory voxel-wise comparison at *p* < 0.001, uncorrected, identified a 28-voxel cluster of increased rCBF in the right cerebellar cortex at follow-up compared with baseline. The peak was located at MNI coordinates (22, −38, −44), with t = 5.96 and z = 3.11 (*p* < 0.001; Table 3 and Figure 2). Because no FWE or FDR correction was applied, this finding should be interpreted as hypothesis-generating.

## 4. Discussion

This exploratory case series examined both clinical outcomes and cerebral perfusion after high-frequency H1-coil dTMS targeting the SMA in PD. Conventional figure-of-eight rTMS can provide relatively focal superficial stimulation, whereas H-coils were designed to produce broader and deeper electric fields at the cost of reduced focality [15,16,17,18]. This broader field profile was the principal rationale for selecting the H1-coil in the present study: the goal was to engage an SMA-centered motor network rather than to directly stimulate a distant subcortical or cerebellar structure. Prior clinical studies have explored H-coil stimulation in PD and have reported variable but potentially meaningful motor effects [4,19,20], while SMA-directed conventional rTMS has also shown effects on bradykinesia and freezing of gait [10,11].

At the three-month follow-up, the exploratory SPECT analysis showed increased right cerebellar perfusion. This finding should be interpreted as a network-level association. The depth of the H1-coil electric field does not support direct electromagnetic stimulation of the cerebellum from an SMA scalp position; instead, SMA stimulation may influence cerebellar physiology through polysynaptic cortico-ponto-cerebellar and cerebello-thalamo-cortical pathways and through broader changes in motor-network connectivity. Neuroimaging studies in PD have demonstrated abnormal cerebellar and motor-network activity, and cerebellar hyperactivation has been interpreted in different studies as potentially compensatory, pathophysiological, or context-dependent [24,25,26,27,28,29,30,31,32]. SMA-directed rTMS has also been shown to alter distributed functional connectivity in PD [33,34,35]. Therefore, the present perfusion finding is biologically plausible as a downstream network effect, but the SPECT data alone cannot establish the direction or mechanism of connectivity change.

The present data do not allow us to distinguish whether the cerebellar hyperperfusion primarily represents dTMS-induced network modulation, recruitment of pre-existing compensatory circuitry, or a combination of both. The earlier wording that favored a direct dTMS effect over compensation was therefore too strong and has been tempered. Non-motor symptoms were assessed using UPDRS Part I and NMSS. At three months, neither UPDRS Part I nor NMSS showed a statistically significant change, and the direction of individual changes was heterogeneous. Dedicated depression, anxiety, and apathy scales and a formal neuropsychological battery were not systematically administered, so the present series cannot determine whether the cerebellar perfusion change was related to specific affective or cognitive domains. Moreover, because patient-level perfusion values from the identified SPECT cluster were not prespecified for extraction and *n* = 5, formal clinical-imaging correlations were not performed. Future studies should combine standardized cognitive and mood/apathy instruments with prespecified imaging biomarkers to test these relationships.

No clinical outcome reached statistical significance in this five-patient series, but the study is severely underpowered for inferential testing. Descriptively, UPDRS Part III and TUG decreased at follow-up, and mean NMSS also decreased, whereas UPDRS Parts I and II showed small mean increases and UPDRS Part IV was unchanged. These changes were heterogeneous at the individual-patient level. The large-magnitude standardized changes for some outcomes in Table 2 should not be overinterpreted because effect-size estimates are unstable in such a small sample. Antiparkinsonian medication regimens and doses were stable throughout the three-month follow-up, reducing confounding from medication changes over time; nevertheless, the exact timing of the last dopaminergic dose relative to clinical testing and SPECT was not standardized and could still influence motor performance and rCBF. Between-patient differences in disease severity and anatomical targeting may also have contributed to the variable responses. Previous studies have reported both positive and null or heterogeneous motor responses to rTMS in PD, depending on target and stimulation parameters [4,7,8,10,11,20].

Several limitations must be emphasized. First, the sample size was extremely small, and there was no sham-controlled group; natural fluctuation, regression to the mean, placebo/expectation effects, and other time-varying factors cannot be excluded. Second, seven clinical outcomes were examined without multiplicity correction, and the analyses were underpowered; consequently, *p*-values and effect sizes are descriptive and cannot establish efficacy. Third, SMA targeting was approximated using scalp-based positioning rather than individualized neuronavigation, introducing interindividual uncertainty in coil-to-cortex correspondence. Fourth, the SPECT analysis used an exploratory voxel-wise threshold of *p* < 0.001 without FWE or FDR correction. Although the observed right cerebellar cluster comprised 28 voxels with a peak t-value of 5.96, the absence of correction for whole-brain multiple comparisons increases the risk of false-positive findings and precludes confirmatory inference. Fifth, although antiparkinsonian medication regimens and doses remained unchanged during follow-up, the exact interval between the last dopaminergic dose and SPECT was not prospectively standardized, so acute medication-state effects on rCBF cannot be excluded [23]. Sixth, UPDRS Part I and NMSS were available, but dedicated depression/anxiety/apathy scales and standardized cognitive testing such as MMSE/MoCA were not systematically obtained, limiting domain-specific non-motor and cognitive interpretation. Finally, no functional-connectivity analysis was performed, so the proposed SMA–cerebellar network mechanism remains inferential. Despite these limitations, the study combines dTMS with perfusion SPECT and provides a hypothesis-generating observation that can inform larger controlled investigations.

## 5. Conclusions

In conclusion, high-frequency H1-coil dTMS targeting the SMA was followed by an exploratory increase in right cerebellar perfusion in five patients with PD, while no motor or non-motor clinical outcome reached nominal statistical significance at three months. The cerebellar finding should be interpreted as a possible downstream network effect rather than direct cerebellar electromagnetic stimulation, and both dTMS-induced modulation and compensatory recruitment remain plausible. Antiparkinsonian medication regimens and doses were unchanged throughout follow-up, although exact dose-to-SPECT timing was not standardized. Given the very small sample, absence of a sham-controlled group, uncorrected imaging threshold, and absence of standardized neuropsychological and dedicated mood/apathy assessments, these observations are preliminary and should not be interpreted as evidence of clinical efficacy. Future studies should use larger samples, sham control, individualized neuronavigation, standardized medication-to-imaging timing, comprehensive motor/non-motor and neuropsychological outcomes, and prespecified FWE- or FDR-corrected imaging analyses.

## Figures and Tables

**Table 1:** Demographic and clinical characteristics of patients with Parkinson’s disease.

	Case 1	Case 2	Case 3	Case 4	Case 5	Mean ± SD	*p*-Value *
**Age, years**	72	65	54	57	68	63.20 ± 7.53	-
**Sex**	male	female	male	female	female	-	-
**LEDD before dTMS (mg/day)**	675	375	225	450	300	405.00 ± 172.66	-
**LEDD at 3 months (mg/day)**	675	375	225	450	300	405.00 ± 172.66	unchanged
**HY stage before dTMS**	2	2.5	1	2	2	1.90 ± 0.55	-
**HY stage after dTMS**	2	2	1	2	2	1.80 ± 0.45	1.000
**UPDRS Part I before dTMS**	0	0	0	0	1	0.20 ± 0.45	-
**UPDRS Part I after dTMS**	0	1	0	3	0	0.80 ± 1.30	0.750
**UPDRS Part II before dTMS**	8	5	2	6	8	5.80 ± 2.49	-
**UPDRS Part II after dTMS**	8	10	2	5	6	6.20 ± 3.03	1.000
**UPDRS Part III before dTMS**	13	22	7	16	16	14.80 ± 5.45	-
**UPDRS Part III after dTMS**	9	16	3	15	16	11.80 ± 5.72	0.125
**UPDRS Part IV before dTMS**	3	2	0	2	0	1.40 ± 1.34	-
**UPDRS Part IV after dTMS**	2	1	0	2	2	1.40 ± 0.89	1.000
**NMSS before dTMS**	7	36	1	21	11	15.20 ± 13.72	-
**NMSS after dTMS**	3	12.5	1	21	15	10.50 ± 8.38	0.750
**TUG before dTMS (s)**	8.51	12.5	6.5	11.1	12.5	10.22 ± 2.64	-
**TUG after dTMS (s)**	7.18	11	6.84	6.87	11.06	8.59 ± 2.23	0.125

Abbreviations: dTMS, deep transcranial magnetic stimulation; HY, Hoehn–Yahr; LEDD, levodopa equivalent daily dose; NMSS, Non-Motor Symptoms Scale; UPDRS, Unified Parkinson’s Disease Rating Scale; TUG, Timed Up and Go; SD, standard deviation. * *p*-values were calculated using two-sided paired Wilcoxon signed-rank tests and are presented descriptively without multiplicity correction. Medication regimens and doses, and therefore LEDD values, were unchanged during the three-month follow-up.

**Table 2:** Descriptive paired clinical changes and standardized effect sizes.

Outcome	Baseline, Mean ± SD	3-Month, Mean ± SD	Mean Change (Post − Pre)	*p*-Value *	Cohen’s dz ^†^
HY stage	1.90 ± 0.55	1.80 ± 0.45	−0.10	1.000	−0.45
UPDRS Part I	0.20 ± 0.45	0.80 ± 1.30	+0.60	0.750	+0.40
UPDRS Part II	5.80 ± 2.49	6.20 ± 3.03	+0.40	1.000	+0.15
UPDRS Part III	14.80 ± 5.45	11.80 ± 5.72	−3.00	0.125	−1.22
UPDRS Part IV	1.40 ± 1.34	1.40 ± 0.89	0.00	1.000	0.00
NMSS	15.20 ± 13.72	10.50 ± 8.38	−4.70	0.750	−0.43
TUG (s)	10.22 ± 2.64	8.59 ± 2.23	−1.63	0.125	−0.99

Abbreviations: HY, Hoehn–Yahr; NMSS, Non-Motor Symptoms Scale; UPDRS, Unified Parkinson’s Disease Rating Scale; TUG, Timed Up and Go. * *p*-values are from two-sided paired Wilcoxon signed-rank analyses and are presented descriptively without multiplicity correction. ^†^ Cohen’s dz was calculated from within-patient change scores. Negative values indicate decreases in the corresponding scale; for HY, UPDRS, NMSS, and TUG, lower scores generally represent less impairment. Effect-size estimates are highly uncertain because *n* = 5.

**Table 3:** Brain region with increased regional cerebral blood flow after deep transcranial magnetic stimulation.

Brain Region	Cluster Size (Voxels)	t	z	*p*-Value	Peak MNI Coordinates *
Right cerebellar cortex	28	5.96	3.11	<0.001	22, −38, −44

* Coordinates refer to the Montreal Neurological Institute (MNI) coordinate system.

## Data Availability

The data presented in this study are available from the corresponding author upon reasonable request, subject to ethical and privacy restrictions.
